# Biosignal processing methods to explore the effects of side-dominance on patterns of bi- and unilateral standing stability in healthy young adults

**DOI:** 10.3389/fphys.2022.965702

**Published:** 2022-09-16

**Authors:** János Négyesi, Bálint Petró, Diane Nabil Salman, Ahsan Khandoker, Péter Katona, Ziheng Wang, Anfal Ibrahim Sanqour Qambar Almaazmi, Tibor Hortobágyi, Márk Váczi, Kristóf Rácz, Zsófia Pálya, László Grand, Rita M. Kiss, Ryoichi Nagatomi

**Affiliations:** ^1^ Division of Biomedical Engineering for Health and Welfare, Tohoku University Graduate School of Biomedical Engineering, Sendai, Japan; ^2^ Faculty of Mechanical Engineering, Department of Mechatronics, Optics and Mechanical Engineering Informatics, Budapest University of Technology and Economics, Budapest, Hungary; ^3^ Biomedical Engineering Department, Khalifa University, Abu Dhabi, United Arab Emirates; ^4^ Department of Kinesiology, Hungarian University of Sports Science, Budapest, Hungary; ^5^ School of Biomedical Engineering, The University of Sydney, Sydney, NSW, Australia; ^6^ Center for Human Movement Sciences, University of Groningen, University Medical Center Groningen, Groningen, Netherlands; ^7^ Somogy County Kaposi Mór Teaching Hospital, Kaposvár, Hungary; ^8^ Department of Sport Biology, Institute of Sport Sciences and Physical Education, University of Pécs, Pécs, Hungary; ^9^ Faculty of Information Technology, Pázmány Péter Catholic University, Budapest, Hungary; ^10^ Department of Medicine and Science in Sports and Exercise, Tohoku University Graduate School of Medicine, Sendai, Japan

**Keywords:** balance, hand dominance, laterality, leg-dominance, motion capture, posture

## Abstract

We examined the effects of side-dominance on the laterality of standing stability using ground reaction force, motion capture (*MoCap*), and *EMG* data in healthy young adults. We recruited participants with strong right (*n* = 15) and left (*n* = 9) hand and leg dominance (side-dominance). They stood on one or two legs on a pair of synchronized force platforms for 50 s with 60 s rest between three randomized stance trials. In addition to 23 *CoP*-related variables, we also computed six *MoCap* variables representing each lower-limb joint motion time series. Moreover, 39 time- and frequency-domain features of *EMG* data from five muscles in three muscle groups were analyzed. Data from the multitude of biosignals converged and revealed concordant patterns: no differences occurred between left- and right-side dominant participants in kinetic, kinematic, or *EMG* outcomes during bipedal stance. Regarding single leg stance, larger knee but lower ankle joint kinematic values appeared in left vs right-sided participants during non-dominant stance. Left-vs right-sided participants also had lower medial gastrocnemius *EMG* activation during non-dominant stance. While right-side dominant participants always produced larger values for kinematic data of ankle joint and medial gastrocnemius *EMG* activation during non-dominant vs dominant unilateral stance, this pattern was the opposite for left-sided participants, showing larger values when standing on their dominant vs non-dominant leg, i.e., participants had a more stable balance when standing on their right leg. Our results suggest that side-dominance affects biomechanical and neuromuscular control strategies during unilateral standing.

## Introduction

Functions such as language, speech, or face recognition show localization to one side of the brain. This phenomenon is called hemispheric lateralization. Due to the evolutionary specialization of the left hemisphere for skilled motor activities (Goodale, 1988; Gonzalez and Goodale, 2009; Stone et al., 2013), 90% of healthy adults are right-hand dominant and perform fundamental manual motor tasks with the right hand (Perelle and Ehrman, 2005; Vuoksimaa et al., 2009; Sartarelli, 2016). This behavioural asymmetry is known as “right-handedness” or “right-hand dominance”. The nature of hand dominance is also a consequence of brain lateralization through complex motor control processes (for reviews, see (Hatta, 2007; Sainburg, 2014)). Specifically, left-handedness is a marker of atypical cerebral lateralization. Unlike the upper limbs, the determination of leg dominance is more complicated because leg vs hand dominance is much less lateralized and seems to be task-dependent (Gabbard and Hart, 1996). Most of the leg dominance tasks are performed under bilateral conditions so that one leg stabilizes the system while the other leg moves as in kicking a ball, stepping up on a chair, or high jumping. The consensus suggests the mobilizing limb is the dominant leg in lower extremity tasks requiring two legs, an idea also supported by neurodevelopmental studies (Pompeiano, 1985; Previc, 1991). These studies suggest that the left leg subserves postural tasks while the right leg concurrently generates voluntary movements. Such functional organization is due to the asymmetric prenatal development of the vestibular function on the left side. Consequently, it may be not surprising that the left leg is suggested to be the preferred limb for tasks of unipedal stability (Maki, 1991). Overall, it seems that right-hand dominant people tend to be right-legged.

Feedback from lower extremity proprioceptors shapes postural stability in standing (Allum et al., 1998). Joint position sense (*JPS*) measurements reveal proprioceptive function at the knee (Barrett et al., 1991) and ankle (Konradsen, 2002) joints. Strongly right-side dominant individuals consistently sense movements more accurately in both upper and lower extremity joints of the non-dominant left vs the right-dominant side (Roy and MacKenzie, 1978; Kurian et al., 1989; Nishizawa, 1991; Goble et al., 2006; Goble and Brown, 2007, 2008; Han et al., 2013; Negyesi et al., 2019). We also found that left-sided participants performed a target-matching task more accurately with their dominant left vs right knee joint (Galamb et al., 2018). These data suggest that right-hemisphere specialization may underlie proprioceptive feedback (Naito et al., 2005; Goble and Brown, 2007, 2008), regardless of hand and leg dominance (side dominance). This idea is supported by clinical data demonstrating postural impairments including the side ipsilateral to a right-hemisphere stroke (Bohannon et al., 1986; Perennou et al., 2008; Duclos et al., 2015). It thus seems that central and peripheral functional asymmetry may differ between left-vs right-side dominant individuals. Therefore, further research is needed to resolve the differences in co-lateralization of postural stability between left- and right-side dominant healthy adults.

Balance is a fundamental motor skill that underlies gait and posture through the activation of muscular synergies (Torres-Oviedo and Ting, 2007; Chvatal and Ting, 2012) generated by the central nervous system (*CNS*) (Winter 1995; Winter et al., 2003). Proprioceptive feedback from the lower extremity joints forms an important element of this control. Static balance is the maintenance of the equilibrium under unperturbed conditions such as during quiet standing (Papegaaij and Hortobágyi, 2017). Standing balance stability can be assessed by multiple ways, including kinetics, kinematics, and neural control via electromyography (*EMG*). During quiet standing, the base of support is fixed and balance is maintained by countering torques produced by the gravitational force around the center of mass (*CoM*). As a result, the center of pressure (*CoP*) shifts within the base of support. Because balance is controlled by keeping the position of the *CoM* between the weight-bearing limbs (Gray, 1944), kinetic data of *CoP* movements provide information on how the *CNS* controls standing stability through the involvement of three synergistic muscle groups (Krishnamoorthy et al., 2004). Short *CoP* paths are associated with high standing stability (Lepers et al., 1997). Whether or not leggedness affects bi- or unipedal standing stability is unclear. *CoP* path does not seem to differ between dominant vs non-dominant leg during unilateral (Matsuda et al., 2008; Lopez-Fernandez et al., 2020) and bipedal (Haddad et al., 2011) stance. Perhaps *CoP* path data are not sensitive enough for detecting leg-differences in standing stability. Especially, because these studies only recruited right-side dominant participants and limited their analysis only to *CoP* data. Such data form a resultant outcome that condenses information on the whole-body posture and information on postural accelerations into one two-dimensional (2D) variable (Federolf, 2016). Nevertheless, examining a variety of traditional *CoP* metrics including sway, velocity, and area of ellipse (Prieto et al., 1996; Rocchi et al., 2004; Sun et al., 2018) is fundamental and important for the assessment of standing stability.

Because athletes often encounter situations in which the *CoM* is controlled while standing on one leg, *CoP* paths and velocity are complementary to *CoM* outcomes in indexing standing stability (Vuillerme et al., 2001; Paillard et al., 2006). A complimentary approach to assessing balance stability is kinematics, using motion capture (*MoCap*). Specifically, by affixing reflective markers over anatomical landmarks, we can measure body kinematics by a motion capture system that determines the positions of the markers (Alradwan et al., 2015). Briefly, several camera recordings are used by the system to calculate trajectories of the markers or the position and orientation of the rigid bodies, and to estimate the motions of the underlying bones to produce joint kinematics data (Cappozzo et al., 2005). Lower limb joint angular kinematics via *MoCap* is widely used in the last few decades to analyze postural control (reviewed in (Roggio et al., 2021)). While *CoP* provides information only about foot-related events, *MoCap* data informs us about *CoM* displacement and other descriptive aspects of standing balance stability. With respect to stability, measuring a set of variables representing change and deviation for each lower extremity joint motion time series, e.g., range, total movement, velocity, or acceleration would shed light on the laterality effects on standing stability.

Ultimately, the neural command controls kinetic and kinematic features by activating muscle groups and muscle synergy modules. Specifically, earlier studies using principal component analysis (PCA) identified that muscles are not independently controlled but work in groups in various tasks requiring shifts of the *CoP* (Krishnamoorthy et al., 2003a; Krishnamoorthy et al., 2003b). Therefore, *EMG* data analyses from five muscles in these three synergistic muscle groups (anterior leg muscles, posterior leg muscles, trunk muscles) responsible for controlling posture (Krishnamoorthy et al., 2004) can provide insights into the neural control of stability while standing (Danna-Dos-Santos et al., 2015). The relationship between standing and walking balance abilities and muscle activation is well described (Papegaaij and Hortobágyi, 2017; Cinthuja et al., 2021). The literature (Phinyomark et al., 2012a; b; Phinyomark et al., 2014; Karthick and Ramakrishnan, 2016; Khushaba et al., 2017; Samuel et al., 2018; Waris and Kamavuako, 2018; Too et al., 2019; Verma and Gupta, 2019; Toledo-Pérez et al., 2020) also reports a wide spectrum of time- and frequency-domain features of the *EMG* signal that could help to determine if the synergistic muscles’ activity contributes to the potential differences, if any, between left- and right-side dominant individuals during bi- and unipedal stance.

It is unclear if the aforementioned three methods produce complementary results when used in combination, a measurement scheme rarely used. Therefore, the primary objective of the present study was to examine whether side-dominance affects the laterality of bi- and unilateral postural stability during quiet standing in healthy younger adults using a multitude of biosignal processing methods: *CoP*, *MoCap*, and *EMG* data**.** Based on the preponderance of data from previous studies suggesting right-hemisphere specialization for standing stability, we hypothesize right-side dominant participants to have more stable standing balance in their non-dominant left vs right-dominant leg. However, left-sided participants may have more stable standing balance in their dominant left as compared to their non-dominant right leg. Consequently, during non-dominant unilateral stance, we expect more stable standing balance in right-compared to left-sided participants but vice versa during dominant leg stance. We also hypothesize that these between-group and within-group differences will be concordantly reflected across measures of kinetics, kinematics, and muscle activation. Our study fits under the current research efforts towards understanding the effects of side-dominance on postural control.

## Methods

### Participants

Sample size calculations (G*Power 3.1.7 (Faul et al., 2007)) based on a previous study (Danna-Dos-Santos et al., 2015) that aimed to determine the effects of visual information on multi-muscle control during quiet stance, revealed that a total sample size of 16 would be appropriate to detect significant differences between the groups, assuming type I error of 0.05 and power of 0.80.

A large number of potential participants were asked to fill out two inventories to clarify their hand and leg dominance. Hand dominance was determined using the Edinburgh Handedness Inventory (Oldfield, 1971), a scale that is used to measure the degree of hand laterality in daily activities such as writing, drawing, throwing, using scissors, brushing teeth, opening a box, striking a match and using a pair of scissors, a knife, a spoon, and a broom. Leg dominance was determined by one- or two-foot item skill tests such as kicking a ball or stepping up on a chair (Spry et al., 1993). Laterality index (LI) for both hand and leg dominance was calculated by summing the number of tasks performed with the right limb (R) and the number of tasks performed with the left limb (L) as follows (R - L)/(R + L). The required level of laterality for both hand and leg dominance was LI ≥ 0.9.

We finally recruited nine left-side dominant (age = 27.9 ± 5.8 years; height = 179.2 ± 7.6 cm; mass = 76.3 ± 8.2 kg; three females) and 15 right-side dominant (age = 28.2 ± 5.5 years; height = 173 ± 8.2 cm; mass = 67.9 ± 13.3 kg; six females) participants who met our laterality conditions with no reported neurological deficit or sensorimotor impairment. Laterality index for both hand and leg dominance was ≥0.9 in right-side dominant, and ≥ -0.9 in left-side dominant participants, showing strong right- or left-side dominance, respectively. After giving both verbal and written explanations of the experimental protocol, participants signed the informed consent document in accordance with the declaration of Helsinki. The study was carried out in accordance with the recommendations of the University Ethical Committee (Approval No. TE-KEB:2:2021).

### Experimental design

Each participant performed barefoot bi- and unilateral (with both the dominant and non-dominant leg) quiet standing trials on either two or one force plate, respectively, both with eyes-open in a randomized order. Participants were asked to stand as still as possible during each trial. Each trial lasted for 50 s with a rest period of 60 s between trials (Garcia-Masso et al., 2016). Each participant adopted the same foot placement and posture during the tasks: 1.) during bipedal stance, the participants’ heels were separated by the width of their shoulders and their toes pointed forward, while 2.) during unilateral stance, participants were asked to raise their heel by flexing their non-supporting leg’s knee joint at 90° to ensure the absence of contact between the foot and the floor during the trial. Only successful trials were considered, therefore, if the participants bent their trunk or arms, or touched down with the non-supporting leg, the trial was repeated after a recovery period of 60 s. Nevertheless, the frequency of failures was very low and did not differ between right and left-side dominant participants neither during dominant nor during non-dominant unilateral stance. During each trial, participants were instructed to look at a point of reference (5 cm in diameter) placed in front of them at eye level at a distance of 2 m and keep their arms down by their side. The *CoP*, *MoCap*, and *EMG* recordings were synchronized in time by starting the data analyses from a trigger signal that appeared in all channels.

### Experimental procedures

#### CoP-related kinetics

Ground reaction force (*GRF*) was measured by a pair of synchronized force platforms (P-6000, BTS Bioengineering SnA., Garbagnate Milanese MI, Italy) at a sampling rate of 1 kHz. The two platforms recorded the *GRF* in a common reference frame that coincided with the *MoCap* system’s reference frame. During bipedal standing, one foot was placed on each platform. The platforms are able to measure the *CoP* (point of attack of the *GRF*), the magnitude of the *GRF* in three dimensions, and the torque around the vertical axis that goes through the *CoP*.

### Kinematics

**FIGURE 1:**
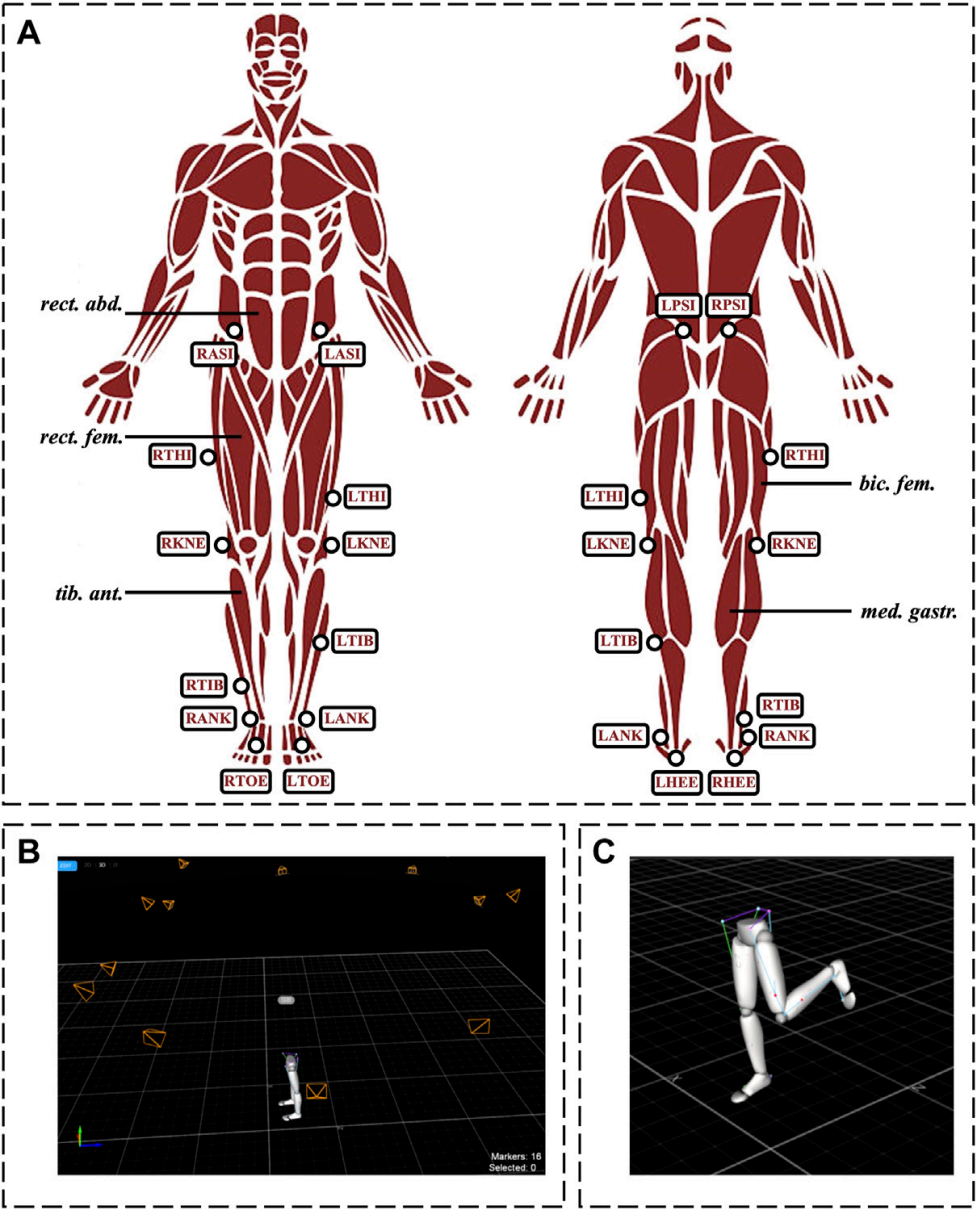
Experimental setup. Panel **(A)**: Schematic illustration of the measured muscles for EMG analysis, and the placement of the reflective markers for motion capture measurements. *Panel* **(B)** *and* **(C)** *:* 3D avatar of a representative subject’s lower limbs during bi- and unilateral stances, respectively. Muscles: bic. fem., biceps femoris; med. gastr., medial gastrocnemius; rect. abd., rectus abdominis; rect. fem., rectus femoris; tib. ant., tibialis anterior. Markers: LANK/RANK, left/right lateral malleolus; LASI/RASI, left/right anterior superior iliac spine; LHEE/RHEE, left/right heel (bisection of the distal aspect of the posterior calcaneum); LKNE/RKNE, left/right knee (lateral epicondyle of the femur); LPSI/RPSI, left/right posterior superior iliac spine; LTHI/RTHI, left/right thigh (not an exact location, only to aid with sides); *LTIB/RTIB*, left/right shank (not an exact location, only to aid with sides); *LTOE/RTOE*, left/right toes (between the distal ends of the 1st and 2nd metatarsi).

In addition, participants’ movements were captured via an optical-based *MoCap* system (OptiTrack, 18 pieces of Flex13 cameras, NaturalPoint Inc., Oregon, United States ) at a 100 fps sampling rate using 16 skin-attached retro-reflective markers placed at anatomical locations (Figure 1A). The 3D avatar of a representative participant during bi- and unilateral stances is shown in Figures 1B,C, respectively.

### Electromyography (EMG)

To record *EMG* signals, a Cometa Wave Plus wireless *EMG* system with Mini Wave Waterproof units and the EMGandMotionTools 7.0.10.0 software (Cometa S. r.l., Bareggio MI, Italy) were used. *EMG* signals were recorded on both sides of the body using surface bipolar electrodes. The measured muscles and muscle groups were the following: 1. Posterior muscles: medial gastrocnemius and biceps femoris, 2. Anterior muscles: tibialis anterior and rectus femoris, 3. rectus abdominis (Figure 1A). Electrodes were placed according to the recommendations of the SENIAM project (seniam.org) on the muscular belly with an inter-electrode distance of approximately 20 mm. The sampling frequency was 2000 Hz.

### Data analyses

#### CoP-related kinetic data

The *CoP*-sway time-series collected were jointly defined by anterior-posterior (*AP*) and medio-lateral (*ML*) direction signals. Considering that the analysis of phase lag or time-related signal features was unnecessary in the present study, the *AP* and *ML* time-series were first filtered at 5 Hz using a 4^th^ order Butterworth low pass filter (Prieto et al., 1996; Sun et al., 2018), then referenced to the mean *CoP*. Furthermore, the resultant distance time series was calculated to quantify the *CoP* signal in the combined *AP* and *ML* directions. The first second of data was excluded from the analysis to eliminate the filter’s transient response. To analyze the *CoP*-sway and assess standing stability, a total of twenty-three variables (Table 1) were computed from both the independent *AP* and *ML* time-series as well as the resultant distance time-series using MATLAB^®^ (MATLAB R2021b, MathWorks, Natick, MA, United States ). A variety of traditional *CoP* metrics often used in literature (Prieto et al., 1996; Rocchi et al., 2004; Sun et al., 2018) were determined from the *CoP* data, including: the mean distance (*mdist*), standard deviation (*SD*), root mean square distance (*RMS_dist*), range (*range*), path length (*pathlength*), mean velocity (*mvelo*), 95% confidence ellipse area (*area_ce*), fractal dimension (*fd*), sway area (*area_sw*) and mean frequency (*mfreq*). Moreover, the sample entropy (*SampEn*) was evaluated to analyze the regularity and complexity of the *CoP*-sway signal. Briefly, *SampEn* is the negative logarithm of the conditional probability that a dataset having repeated itself for *m* samples within a tolerance *r* will also repeat itself for *m+1* samples, excluding self-matches. The *SampEn* algorithm was obtained from Richman et al. (Richman and Moorman, 2000). A range of *m* values from 2 to 9 and *r* values from 0.01 to 0.9 were examined and the parameters *m* = 2 and *r* = 0.01 were found to be appropriate for our data. The signal length was fixed at *N* = 24,000 samples for all the *SampEn* computations and the total sample entropy was determined as the average of *SampEn* results for consecutive signal intervals of size *N* starting from the end of the signal. Table 1 presents and defines all the *CoP*-based variables computed in this study. To control for weight distribution during bipedal stance, the symmetry index (Rougier and Genthon, 2009; Hendrickson et al., 2014) was calculated using the total *GRF* measured under each foot from the left and right force platforms.

$$\text{Symmetry}\;\text{index}=\frac{Dominant\;limb\;value}{Non-dominant\;limb\;value+Dominant\;limb\;value}$$

**TABLE 1:** Variables computed for *CoP* analysis

Description of CoP data obtained from force plate					
Time series	Abbreviation		Unit	Description	Equation
Raw CoP time series (AP, ML)	AP0; ML0		mm	Time series describing the CoP path relative to the origin of the force platform in the anterior-posterior (AP; z-axis) and medial-lateral (ML; x-axis) directions	N.A. (directly recorded from force plate)
Signal filtered using 4th order Butterworth low pass filter with 5 Hz cutoff frequency (Prieto et al., 1996; Sun et al., 2018) First second (=1000 samples) of signal cut out to eliminate the filter transient effect.					
CoP time series referenced to mean (AP, ML)	AP; ML		Mm	CoP path time series describing the change of CoP relative to the mean CoP value	AP= AP0 - mean(AP0)
					ML= ML0 - mean(ML0)
Resultant Distance time series (comb. AP and ML)	Rd		Mm	Resultant distance time series describing vector distance from mean CoP to pair of points in AP0 and ML0	$rd=\surd \left(AP^{2}+ML^{2}\right)$
Traditional CoP measures (Prieto et al., 1996)					
Variable name	Abbreviation		Unit	Description	Equation
Fractal dimension - 95% Confidence Ellipse	Fd		-	Fractal dimension is a unitless measure of the degree to which a curve fills the metric space which it encompasses. Fractal dimension confidence ellipse models the area of the stabilogram with the 95% confidence ellipse	$fd=\log\left(N\right)/\log\left(\frac{N*d}{pathlength}\right)$
					d=√(2a*2b)
Mean Distance	Mdist		Mm	Mean resultant distance; average distance from the mean CoP	mdist=mean(rd)
Mean Distance AP	mdist_AP		Mm	Average AP distance from mean CoP	mdist_AP=mean(|AP|)
Mean Distance ML	mdist_ML		Mm	Average ML distance from mean CoP	mdist_ML=mean(|ML|)
Mean Frequency	mfreq		Hz	Mean frequency is the rotational frequency, in revolutions per second or Hz, of the CoP if it had traveled the total excursions around a circle with a radius of the mean distance	$mfreq=\frac{mvelo}{2\pi *mdist}$
Mean Velocity	mvelo		mm/s	Average velocity of resultant CoP	$mvelo=\frac{pathlength}{T}$
Mean Velocity AP	mvelo_AP		mm/s	Average velocity of CoP path in the AP direction	$mvelo\_AP=\frac{pathlength\_\text{AP}}{T}$
Mean Velocity ML	mvelo_ML		mm/s	Average velocity of CoP path in the ML direction	$mvelo\_ML=\frac{pathlength\_\text{ML}}{T}$
Path Length	pathlength		Mm	Total length of resultant distance CoP path	$pathlength=\sum \surd \left[{\left(AP\left(n+1\right)-AP\left(n\right)\right)}^{2}+{\left(ML\left(n+1\right)-ML\left(n\right)\right)}^{2}\right]$
Path Length AP	pathlength_AP		Mm	Total length of CoP path in the AP direction	pathlength_AP=∑|AP(n+1)−AP(n)|
Path Length ML	pathlength_ML		Mm	Total length of CoP path in the ML direction	pathlength_ML=∑|ML(n+1)−ML(n)|
Range	Range		Mm	Maximum distance between any 2 points on the CoP resultant distance	range=|max(rd)−min(rd)|
Range AP	range_AP		Mm	Maximum distance between any 2 points on the CoP path in the AP direction	range_AP=|max(AP)−min(AP)|
Range ML	range_ML		Mm	Maximum distance between any 2 points on the CoP path in the ML direction	range_ML=|max(ML)−min(ML)|
Root Mean Square distance	RMS_dist		Mm	RMS distance from mean CoP for resultant distance time series	$RMS\_dist=\surd \left(\frac{1}{N}\sum rd^{2}\right)$
Standard Deviation	SD		Mm	Standard deviation of resultant distance (rd) time series	$SD=\surd \left(\frac{1}{N}\sum {\left(rd-mdist\right)}^{2}\right)$
Standard Deviation AP	SD_AP		Mm	Standard deviation of AP time series	$SD\_AP=\surd \left(\frac{1}{N}\sum AP^{2}\right)$
Standard Deviation ML	SD_ML		Mm	Standard deviation of ML time series	$SD\_ML=\surd \left(\frac{1}{N}\sum ML^{2}\right)$
Sway Area	area_sw		mm^2^/s	Sway area estimates the area enclosed by the CoP path per unit of time. Approximated by summing the area of the triangles formed by two consecutive points on the CoP path and the mean CoP	$area\_sw=\frac{1}{2T}\sum \left|AP\left(n+1\right)ML\left(n\right)-AP\left(n\right)ML\left(n+1\right)\right|$
95% Confidence Ellipse Area	area_ce		mm^2^	The 95% confidence ellipse area is the area of the 95% bivariate confidence ellipse, which is expected to enclose approximately 95% of the points on the resultant distance CoP path	${\mathrm{area}}_{\mathrm{ce}}=\pi ab$
					$a=\surd \left(F_{0.5\left[2,n-2\right]}\left(SD_{AP}^{2}+SD_{ML}^{2}+D\right)\right)$
					$b=\surd \left(F_{0.5\left[2,n-2\right]}\left(SD_{AP}^{2}+SD_{ML}^{2}-D\right)\right)$
					$D=\surd \left[\left(SD_{AP}^{2}+SD_{ML}^{2}\right)-4\left(SD_{AP}^{2}SD_{ML}^{2}-SD_{APML}^{2}\right)\right]$
					$SD_{APML}=\frac{1}{N}\sum AP\left(n\right)ML\left(n\right)$

Entropy measures					
Variable name	Abbreviation	Unit		Description	Equation
Sample Entropy	SampEn	-		Measure of complexity and regularity of the magnitude of the resultant displacement time series	SampEn is defined as the logarithmic likelihood that the patterns of the data that are close to each other will remain close for the next comparison within a longer pattern. Algorithm from (Richman and Moorman, 2000).
Sample Entropy AP	SampEn_AP	-		Measure of complexity and regularity of the AP time series	Briefly: A template of defined length, **m**, is created from the first few data point in the time series. The template is then compared to successive data points in the time series and a match is counted if it is within a defined threshold, **r**, of the template.
Sample Entropy ML	SampEn_ML	-		Measure of complexity and regularity of the ML time series	Here, **m = 2** and **r = 0.01**

The symmetry index can range between 0 and 1. A symmetry index of 0.5 indicates that the values for the dominant and non-dominant limbs are equal, i.e., represents perfect symmetry. If the index is greater than 0.5, the dominant vs. non-dominant limb has a greater value of weight distribution during bipedal stance.

### Kinematic data acquired from MoCap analysis

Experimental marker placement was replicated on a lower limb model in OpenSim 4.3 (Delp et al., 2007; Seth et al., 2018), a software for biomechanical modeling, simulation and analysis of movement. The model was scaled for each subject and inverse kinematics were solved to determine the joint angles describing the motion in each trial. The rotational movements calculated were the pelvic list, tilt and rotation, hip flexion, rotation and adduction, and knee and ankle flexion. Pelvic translational motion was also included in the analysis. The joint motion time series were filtered using a 4rth order Butterworth low pass filter with a cut-off frequency of 5 Hz (Schurr et al., 2017). Moreover, the first second of all signals was excluded to eliminate the filter’s transient response. To evaluate standing stability, a set of variables representing change and deviation for each joint motion time series were computed in MATLAB^®^ (MATLAB R2021b, MathWorks, Natick, MA, United States ), including: the standard deviation (*sd*), range (*range*), total movement (*totmov*), root mean square velocity (*rvelo*), root mean square acceleration (*raccel*), and mean percentage index (*mpi*). The motion capture analysis variables used in this study are summarized and defined in Table 2.

**TABLE 2:** Variables computed for MoCap analysis

Motion time series obtained from Inverse Kinematics				
Motion time series	Abbreviation	Unit	Description	
Pelvis list	pelvis_list	degrees	Pelvis forward-backward rotational motion	
Pelvis tilt	pelvis_tilt	degrees	Pelvis right-left rotational motion	
Pelvis rotation	pelvis_rotation	degrees	Pelvis internal-external rotation	
Pelvis medial-lateral translation	pelvis_tx	m	Pelvis right-left translation	
Pelvis anterior-posterior translation	pelvis_ty	m	Pelvis forward-backward translation	
Pelvis cranial-caudal translation	pelvis_tz	m	Pelvis upward-downward translation	
Hip flexion	hip_flexion	degrees	Hip forward-backward rotational motion	
Hip rotation	hip_rotation	degrees	Hip internal-external rotation	
Hip adduction	hip_adduction	degrees	Hip right-left rotational motion	
Knee flexion	knee_angle	degrees	Knee forward-backward rotational motion	
Ankle flexion	ankle_angle	degrees	Ankle upward-downward rotational motion	

Variables computed for each motion time series (y) for MoCap analysis				
Variable name	Abbreviation	Unit	Description	Equation
Standard deviation	Sd	Degrees m	Standard deviation of the rotational or translational joint motion time series	$sd=\surd \left(\frac{1}{N}\sum {\left(y-mean\left(y\right)\right)}^{2}\right)$
Range	Range	Degrees m	Maximal amplitude of rotational movement or translational movement: maximum distance between any 2 points of the motion time series	range=|max(y)−min(y)|
Total movement	Totmov	Degrees m	Total length of time series representing total rotational or translational movement	$totmov=\sum \surd \left[{\left(y\left(n+1\right)-y\left(n\right)\right)}^{2}\right]$
RMS velocity	Rvelo	degrees/s m/s	Root mean square velocity of rotational or translational motion	$rvelo=\surd \left(\frac{1}{N}\sum {\left(\frac{dy}{dt}\right)}^{2}\right)$
RMS acceleration	Raccel	degrees/s^2^ m/s^2^	Root mean square acceleration of rotational or translational motion	$raccel=\surd \left(\frac{1}{N}\sum {\left(\frac{d^{2}y}{dt^{2}}\right)}^{2}\right)$
Mean percentage index	Mpi	%, deg %, m	Average percent change in position from base position	$PI=\frac{y\left(n+1\right)-y\left(n\right)}{y\left(n\right)}*100\%$
				mpi=mean(PI)

### EMG data

The *EMG* data from all 10 channels was obtained from five different muscles of both the left and right lower limbs during each standing condition. First, wavelet filters were applied using MATLAB^®^ (Misiti et al., 1996) to remove any unwanted artifacts in the signal obtained. In this study, we used wavelet filters between 30 and 500 Hz to reduce the effects of high-frequency noise and low-frequency artifacts in the EMG signals. In MATLAB ‘wfilters ( )’ returns the four lowpass and highpass, decomposition, and reconstruction filters associated with the orthogonal or biorthogonal wavelet. In order to set the cut-off frequencies to make the custom wavelet filter, we decomposed the *EMG* signals into 13 levels and reconstructed from levels 2 to 5 of decomposed signals with symlet mother wavelet (Sym5). More details can be found elsewhere (Vidakovic and Mueller, 1994). Following that, 39 different time- and frequency-domain features were extracted from each channel of the filtered signal. A brief description of the examined *EMG* features, their abbreviations, the corresponding equations, and the relevant references are shown in Table 3.

**TABLE 3:** Variables computed for EMG analysis

Time-domain features			
Variable name	Abbreviation	Description	Equation
3^rd^ Temporal Moment	TM3	Absolute value of cubed EMG signal	$TM3=\left|\frac{1}{k}\overset{k}{\underset{n=1}{\sum }}x_{n}^{3}\right|$
Absolute Value of Summation of Square Root	ABS_SQRT	Motion identification	$ABS\_SQRT=\left|\overset{k}{\underset{n=1}{\sum }}\sqrt{\left(x_{n}\right)}\right|$
			where k is the analysis window
Absolute Value of Summation of Exponential Root	ABS_EXP	Provides insight on the amplitude of the signal	$ABS\_\mathrm{EXP}=\left|\frac{\sum_{n=1}^{k}{\left(x_{n}\right)}^{\exp}}{k}\right|$
			where exp = 0.5, if ( n ≥ 0.25*k && n ≤ 0.75), else exp = 0.75. and k is the signal length
Average Amplitude Change	AAC	Average of the wavelength measurement	$AAC=\frac{1}{k}\overset{k-1}{\underset{n=1}{\sum }}\left|x_{n+1}-x_{n}\right|$
			where k is the signal length
Cardinality	CARD	Number of unique values within a set	Step 1: $y_{n}=sort\left(x_{n}\right)$
			Step 2: $CARD=\overset{k-1}{\underset{n=1}{\sum }}\left|y_{n}-y_{n+1}\right|>\epsilon$
			where ϵ = 0.01
Difference Absolute Mean Value	DABS_M	Modified MAV; estimated signal amplitude	$DABS\_M=\frac{1}{k}\overset{k-1}{\underset{n=1}{\sum }}\left|x_{n+1}-x_{n}\right|$
Difference Absolute Standard Deviation Value	DABS_SD	Modified standard deviation of the wavelength	$DABS\_SD=\sqrt{\frac{1}{k-1}\overset{k-1}{\underset{n=1}{\sum }}{\left(x_{n+1}-x_{n}\right)}^{2}}$
Difference Variance Value	DVAR	Modified VAR of EMG	$DVARV=\frac{1}{k-2}\overset{k-1}{\underset{n=1}{\sum }}{\left(x_{n+1}-x_{n}\right)}^{2}$
Enhanced Wavelength	ENH_WL	Enhanced wavelength	$ENH\_WL=\overset{k}{\underset{n=2}{\sum }}\left|{\left(x_{n}-x_{n-1}\right)}^{p}\right|$
			where p=(0.75,ifi≥0.2ki≤0.8k)orp=(0.5)
Enhanced Mean Absolute Value	ENH_MAV	Enhanced MAV	$ENH\_MAV=\frac{1}{k}\overset{k}{\underset{n=1}{\sum }}\left|x_{n}^{p}\right|$
			where p=(0.75,ifi≥0.2ki≤0.8k)orp=(0.5)
Integrated EMG	INT_EMG	Summation of absolute value of signal amplitude	$INT\_EMG=\overset{k}{\underset{n=1}{\sum }}\left|x_{n}\right|$
			where k is the signal length
Kurtosis	KURT	Statistical technique represent sharpness of distribution curve	$KURT=\frac{\mu_{4}}{\sigma^{4}}$
			where µ_4_ is the forth central moment, and is standard deviation
Log Coefficient of Variation	LOG_CoV	Logarithmic transformation of CoV	$\mathrm{LOG}\_CoV=\log\left(\frac{\sigma }{\mu }\right)$
Log Detector	LOG	Estimate of muscle contraction force	$\mathrm{LOG}=e^{\frac{1}{k}\sum_{n=1}^{k}\log\left(\left|x_{n}\right|\right)}$
Log Difference Absolute	LOG_DABS_M	Logarithmic transformation of DABS_M	$\mathrm{LOG}\_DABS\_M=\log\left(\frac{1}{k}\overset{k-1}{\underset{n=1}{\sum }}\left|x_{n+1}-x_{n}\right|\right)$
Mean Value			
Log Difference Absolute Standard Deviation Value	LOG_DABS_SD	Logarithmic transformation of DABS_SD	$\mathrm{LOG}\_DABS\_SD=\log\left(\sqrt{\frac{1}{k-1}\overset{k-1}{\underset{n=1}{\sum }}{\left(x_{n+1}-x_{n}\right)}^{2}}\right)$
Log Teager Kaiser Energy Opertor	LOG_TK_EO	Measures instantaneous change in energy	$\mathrm{LOG}\_TK\_EO=\log(\overset{k-2}{\underset{n=1}{\sum }}\left(x_{n}^{2}-\left(x_{n-1}\times x_{n+1}\right)\right)$
Maximum Fractal Length	MFL	Measures low level muscle activation	$MFL=log_{10}\left(\sqrt{\overset{k-1}{\underset{n=1}{\sum }}{\left(x_{n+1}-x_{n}\right)}^{2}}\right)$
Mean Absolute Deviation	MAD	Variation between real assessment and mean assessment	$MAD=\frac{1}{k}\left(\overset{k}{\underset{n-1}{\sum }}\left|x_{n}-\bar{x}\right|\right)$
Mean Absolute Value	MAV	Average of absolute value of signal amplitude	$MAV=\frac{1}{k}\overset{k}{\underset{n=1}{\sum }}\left|x_{n}\right|$
Mean Value of Square Root	M_SQRT	Measure to estimate the total amount of activity in analysis window	$M\_SQRT=\frac{1}{k}\overset{k}{\underset{n=1}{\sum }}\sqrt{x_{n}}$
			where k is the signal length
Modified Mean Absolute Value	MAV1	Extension of MAV; w_n_ is added for robustness	$MAV1=\frac{1}{k}\overset{k}{\underset{n=1}{\sum }}w_{n}\left|x_{n}\right|$
			where w_n_ = 1, if 0.25*k ≤ n ≤ 0.75*k else, w_n_ = 0.5
Modified Mean Absolute Value 2	MAV2	Improvements on MAV1; smoothness of robustness function	$MAV2=\frac{1}{k}\overset{k}{\underset{n=1}{\sum }}w_{n}\left|x_{n}\right|$
			where w_n_ = 1, if 0.25*k ≤ n ≤ 0.75*k
			else w_n_ = , if n < 0.25*k
			else w_n_ = , otherwise
Myopulse Percentage Rate	MYOP	Average value of myopulse output	$MYOP=\frac{1}{k}\overset{k}{\underset{n=1}{\sum }}\left[f\left(x_{n}\right)\right]$
			where f(x) = 1 if x ≥ threshold, or f(x) = 0, otherwise. And threshold = 0.016
New Zero Crossing	NEW_ZC	Zero Crossing with improved threshold	$NEW\_ZC=\overset{k-1}{\underset{n=1}{\sum }}f\left(x_{i},x_{i+1}\right)$
			where $f\left(x_{i},x_{x+1}\right)=\left(1,ifx_{i}>Tandx_{i+1}<Torifx_{i}<Tandx_{i+1}>T\right)$ , otherwise $f\left(x_{i},x_{x+1}\right)=0$
			and where $T=4\left(\frac{1}{10}\overset{10}{\underset{n=1}{\sum }}x_{n}\right)$
Root Mean Square	RMS	Root mean square of the signal	$RMS=\sqrt{\frac{1}{k}\overset{k}{\underset{n=1}{\sum }}x_{n}^{2}}$
Simple Square Integral	SSI	Energy index or the summation of squared signal amplitude	$SSI=\overset{k}{\underset{n=1}{\sum }}x_{n}^{2}$
Skewness	SKEW	Skewness of the signal	$SKEW=\frac{\sum_{n=1}^{k}{\left(x_{n}-\bar{x}\right)}^{3}}{k-1\times \sigma^{3}}$
Slope Sign Change	SSC	Number of times the signal changes between -ve and +ve slopes	$SSC=\overset{k-1}{\underset{n=1}{\sum }}\left[f\left[\left(x_{i}-x_{i-1}\right)\times \left(x_{i}-x_{i+1}\right)\right]\right]$
			where f(x) = 1, if x ≥ threshold, or f(x) = 0, otherwise. And threshold = 0.01
Standard Deviation	SD	Standard deviation of the signal	$SD=\sqrt{\frac{\sum_{n=1}^{k}{\left(x_{n}-\mu \right)}^{2}}{k}}$
Variance	VAR	Variance of the signal	$VAR=\frac{1}{k-1}\left(\overset{k}{\underset{n-1}{\sum }}{\left(x_{n}-\bar{x}\right)}^{2}\right)$
Variance of EMG	VAR_EMG	Power index	$VAR\_EMG=\frac{1}{k-1}\overset{k}{\underset{n=1}{\sum }}x_{n}^{2}$
V-Order	V_O	Non-linear detector; similar in definition to RMS	$V\_O={\left(\frac{1}{k}\overset{k}{\underset{n=1}{\sum }}x_{n}^{v}\right)}^{\frac{1}{v}}$
			where V = 2
Waveform Length	WL	Measure of signal complexity; defined as cumulative length of waveform	$WL=\overset{k-1}{\underset{n=1}{\sum }}\left|x_{n+1}-x_{n}\right|$
Willison Amplitude	W_AMP	Related to the firing of motor unit action potential	$W\_AMP=\overset{k-1}{\underset{n=1}{\sum }}\left[f\left(\left|x_{i}-x_{i+1}\right|\right)\right]$
			where f(x) = 1, if x ≥ threshold, or f(x) = 0, otherwise. And threshold = 0.01
Zero Crossing	ZC	Number of times the amplitudes crosses zero amplitude level	$ZC=\overset{k-1}{\underset{n=1}{\sum }}\left[sgn\left(x_{n}\times x_{n+1}\right)\cap \left|x_{n}-x_{n-1}\right|\geq threshold\right]$
			where sgn(x) = 1, if x ≥ threshold , or sgn(x) = 0, otherwise. And threshold = 0.01

Frequency-domain features			
Variable name	Abbreviation	Description	Equation
Avarage Energy	AVE_E	Mean of the squared signal	$AVE\_E=\bar{\left(x^{2}\right)}$
Interquartile Range	IQR	Divergence regarding 75th and 25th percentile of the group	$IQR=Q_{3}-Q_{1}$
			where Q_1_ refers to first quartile, and Q_3_ refers to third quartile

Dimensionless feature			
Variable name	Abbreviation	Description	Equation
Coefficient of variation	CoV	Quantifies the degree of variability of the signal with respect to mean	$CoV=\frac{\sigma }{\mu }$
			where µ is the mean of the signal, is standard deviation

### Statistical analyses

Statistical analyses were performed using SPSS Statistics Package (version 22.0, SPSS Inc., Chicago, IL, United States ). Variables were normally distributed, measured by Shapiro–Wilk’s test of normality and visual inspection of their histograms. Separate multivariate analysis of variance (*MANOVA*) was applied to comprehensively assess the interaction and/or main effect of 1) *CoP*-related kinetic (dimensionless parameters, parameters for *AP* direction, parameters for *ML* direction), 2) kinematic (hip adduction, hip flexion, hip rotation, knee, ankle), and 3) *EMG* (medial gastrocnemius, biceps femoris, tibialis anterior, rectus femoris, rectus abdominis) variables for the group (side dominance: right-side dominant [*R*], left-side dominant [*L*]) by the laterality (dominant leg, non-dominant leg) during bi- and unilateral standing. In case of significant main effect or interaction, a series of group × laterality mixed *ANOVA* and planned post-hoc tests with Bonferroni correction for multiple comparisons were performed in each condition (bipedal stance, single leg stance) for each dependent measures (*CoP*, *EMG*, *MoCap*) to statistically investigate the effect of side-dominance on the laterality of bi- and unilateral standing stability. The Greenhouse–Geisser correction was used when data violated the assumption of sphericity. Complementary post-hoc analyses (paired-samples t-tests) were used when indicated. Cohen’s effect size (*d*) was also computed as appropriate. Additionally, effect sizes of repetition factors were expressed using partial eta squared (η_p_^2^) (Peat et al., 2008). In order to determine if between- or within-group differences in *CoP*, *EMG*, and *MoCap* data were associated with each other, Pearson’s correlations were computed. Statistical significance was set at *p* < 0.05.

## Results

### Bipedal stance

Table 4, Table 5, Table 6 summarize the results of kinetic, kinematic, and *EMG* data, respectively. The symmetry index analyses showed that none of the participants stood symmetrically during bipedal stance. Specifically, 87% of *L* had greater weight-bearing on their dominant left leg (symmetry index >0.5), while 87% of *R* had greater weight-bearing on their non-dominant left leg (symmetry index <0.5). In other words, 62.5% of participants had greater weight-bearing on their left leg, regardless of side-dominance. Multivariate *ANOVA* (*MANOVA*) demonstrated that there was no significant group and laterality main effects or their interactions (all *p* > 0.05) on *CoP*-related kinetic parameters during bipedal stance, regardless of the dimension of the parameters (dimensionless, *AP* direction, *ML* direction) suggesting that *CoP*-related kinetic data of *L* and *R* was similar both in their dominant and non-dominant leg (Table 4). Furthermore, no group and laterality main effects or their interactions occurred in the kinematic data or EMG activity of the measured muscles during bipedal stance (all *p* > 0.05).

**TABLE 4:** Results of CoP-related kinetic data obtained from force plate

Parameter	Bipedal stance				Dominant leg stance		Non-dominant leg stance	
	L		R		L	R	L	R
	D	ND	D	ND	D	D	ND	ND
Fd	1.37 (0.04)	1.37 (0.06)	1.35 (0.06)	1.36 (0.06)	1.52 (0.03)	1.53 (0.04)	1.51 (0.06)	1.54 (0.04)
mdist (mm)	3.17 (1.09)	3.03 (1.29)	3.79 (0.88)	3.50 (1.49)	8.05 (1.67)	8.62 (2.33)	7.26 (2.81)	8.36 (1.94)
mdist_AP (mm)	3.04 (1.06)	2.94 (1.28)	3.69 (0.83)	3.40 (1.50)	6.02 (1.60)	6.33 (2.00)	5.32 (2.25)	6.19 (1.72)
mdist_ML (mm)	0.76 (0.34)	0.64 (0.28)	0.76 (0.47)	0.61 (0.37)	4.18 (0.82)	4.64 (1.14)	3.81 (1.64)	4.36 (1.10)
mfreq (Hz)	0.26 (0.05)	0.26 (0.10)	0.23 (0.08)	0.24 (0.08)	0.61 (0.11)	0.66 (0.17)	0.58 (0.16)	0.67 (0.14)
mvelo (mm/sec)	4.98 (1.56)	4.59 (1.47)	5.31 (1.65)	4.76 (1.38)	30.94 (7.42)	35.01 (11.18)	26.99 (10.84)	34.54 (8.54)
mvelo_AP (mm/sec)	4.77 (1.52)	4.40 (1.46)	5.08 (1.53)	4.57 (1.32)	20.41 (5.15)	21.58 (8.66)	16.83 (7.12)	20.64 (5.79)
mvelo_ML (mm/sec)	1.26 (0.36)	1.08 (0.44)	1.26 (0.83)	1.00 (0.68)	19.09 (6.27)	23.26 (5.95)	17.48 (7.94)	23.63 (5.81)
pathlength (mm)	244.10 (76.29)	224.75 (71.79)	260.18 (80.62)	233.20 (67.47)	1515.96 (363.51)	1605.90 (305.06)	1322.45 (531.00)	1692.28 (418.53)
pathlength_AP (mm)	233.58 (74.65)	215.58 (71.43)	248.92 (74.84)	223.96 (64.89)	999.94 (252.56)	978.38 (206.57)	824.57 (348.76)	1011.39 (283.52)
pathlength_ML (mm)	61.94 (17.73)	52.77 (21.64)	61.88 (40.66)	48.83 (33.36)	935.24 (307.23)	1079.92 (224.62)	856.56 (389.17)	1158.02 (284.61)
range (mm)	11.25 (3.81)	10.33 (4.92)	12.88 (4.21)	11.22 (4.23)	28.38 (16.53)	24.03 (6.46)	19.79 (6.71)	27.24 (10.16)
range_AP (mm)	19.29 (6.56)	17.87 (7.62)	21.61 (6.33)	19.64 (8.15)	46.56 (20.44)	41.46 (11.98)	34.54 (12.56)	44.99 (16.80)
range_ML (mm)	4.85 (1.60)	3.89 (1.38)	4.54 (2.61)	3.75 (2.22)	29.99 (4.58)	29.76 (6.47)	27.16 (9.82)	31.43 (7.60)
RMS_dist (mm)	3.92 (1.32)	3.70 (1.52)	4.61 (1.01)	4.21 (1.74)	9.23 (1.84)	9.75 (2.70)	8.22 (3.06)	9.56 (2.36)
SD (mm)	2.30 (0.80)	2.11 (0.85)	2.61 (0.63)	2.32 (0.95)	4.46 (1.05)	4.56 (1.39)	3.81 (1.30)	4.63 (1.41)
SD_AP (mm)	3.80 (1.28)	3.60 (1.52)	4.49 (0.97)	4.12 (1.74)	7.55 (1.87)	7.82 (2.49)	6.57 (2.66)	7.74 (2.28)
SD_ML (mm)	0.97 (0.39)	0.79 (0.33)	0.94 (0.56)	0.74 (0.45)	5.23 (0.99)	5.75 (1.39)	4.79 (1.94)	5.49 (1.34)
area_ce (mm^2^/s)	157.0 (98.5)	145.1 (128)	205.6 (93.6)	190.6 (152.9)	1102.9 (463.2)	1293.4 (703.7)	959.5 (556.1)	1219.3 (613.6)
area_sw (mm^2^)	1.17 (0.67)	0.88 (0.40)	1.05 (0.31)	1.09 (0.57)	86.43 (42.44)	109.13 (55.22)	71.55 (38.67)	104.13 (47.02)
SampEn	0.31 (0.05)	0.30 (0.08)	0.33 (0.07)	0.29 (0.06)	0.49 (0.02)	0.50 (0.03)	0.46 (0.09)	0.50 (0.03)
SampEn_AP	0.28 (0.05)	0.27 (0.07)	0.28 (0.06)	0.27 (0.05)	0.45 (0.02)	0.45 (0.03)	0.42 (0.08)	0.44 (0.02)
SampEn_ML	0.08 (0.02)	0.07 (0.03)	0.09 (0.06)	0.07 (0.05)	0.45 (0.02)	0.46 (0.03)	0.40 (0.14)	0.46 (0.02)

Values are mean (SD) of each variables

AP, anterior-posterior direction; D, dominant leg; L, left-side dominant participants; ML, medial-lateral direction; ND, non-dominant leg; R, right-side dominant participants

fd, Fractal dimension - 95% Confidence Ellipse; mdist, Mean Distance; mdist_AP, Mean Distance in AP direction; mdist_ML, Mean Distance in ML direction; mfreq, Mean Frequency; mvelo, Mean Velocity; mvelo_AP, Mean Velocity in AP direction; mvelo_ML, Mean Velocity in ML direction; pathlength, Path Length; pathlength_AP, Path Length in AP direction; pathlength_ML, Path Length in ML direction; range, Range; range_AP, Range in AP direction; range_ML, Range in ML direction; RMS_dist, Root Mean Square distance; SD, Standard Deviation; SD_AP, Standard Deviation in AP direction; SD_ML, Standard Deviation in ML direction; area_sw, Sway Area 95% Confidence; area_ce, Ellipse Area; SampEn, Sample Enthropy; SampEn_AP, Sample Enthropy in AP direction; SampEn_ML, Sample Enthropy in ML direction

For further details on the analyzed parameters, please see Table 1.

**TABLE 5:** Results of kinematic data obtained from MoCap analysis

Parameter	Bipedal stance				Dominant leg stance		Non-dominant leg stance	
	L		R		L	R	L	R
	D	ND	D	ND	D	D	ND	ND
KNEE								
sd (degrees)	0.28 (0.37)	0.14 (0.07)	0.20 (0.06)	0.27 (0.37)	1.75 (0.90)*†	0.84 (0.63)*	0.72 (0.37)†	1.58 (1.33)
range (degrees)	1.41 (1.56)	0.72 (0.31)	1.01 (0.33)	1.19 (1.60)	7.76 (2.92)*†	3.91 (2.38)*	3.69 (1.86)†	8.85 (6.75)
totmov (degrees)	22.63 (15.66)	16.43 (5.82)	24.56 (13.60)	28.22 (41.97)	120.64 (53.94)*†	71.80 (36.98)*	71.03 (25.83)†	138.27 (73.89)
rvelo (degrees/s)	0.62 (0.42)	0.44 (0.16)	0.66 (0.37)	0.73 (1.10)	3.45 (1.62)*†	2.09 (1.08)*	1.95 (0.72)†	4.55 (3.11)
raccel (degrees/s^2^)	14.95 (11.58)	10.74 (4.37)	16.55 (9.67)	19.29 (29.09)	58.35 (32.71)†	38.70 (21.62)	34.13 (12.28)†	83.44 (59.92)
ANKLE								
range (degrees)	1.03 (0.51)	0.83 (0.34)	1.00 (0.24)	1.11 (0.50)	3.66 (1.14)†	2.74 (1.21)†	2.78 (1.60)*†	4.57 (2.43)*†
totmov (degrees)	23.57 (7.19)	17.32 (7.60)	20.21 (9.94)	24.17 (15.94)	72.70 (28.59)†	56.50 (19.67)†	50.61 (21.33)*†	89.49 (38.13)*†
rvelo (degrees/s)	0.63 (0.20)	0.45 (0.20)	0.55 (0.26)	0.63 (0.42)	2.05 (0.84)†	1.73 (0.64)†	1.40 (0.60)*†	2.65 (1.24)*†
raccel (degrees/s^2^)	17.30 (6.08)	12.06 (6.07)	14.00 (7.69)	16.90 (11.83)	37.77 (14.89)†	33.32 (13.85)†	24.36 (10.43)*†	52.36 (25.61)*†

* between-group differences during dominant/non-dominant leg stance (p < 0.05); † within-group differences between dominant and nondominant leg stance (p < 0.05)

For further details on the analyzed parameters, please see Table 2.

Values are mean (SD) of each variables. Only those MoCap variables are included in which significant difference were found between or within the groups during bi- or unilateral stance.

Range; raccel, RMS acceleration; rvelo, RMS velocity; sd, Standard Deviation; totmov, Total movement.

**TABLE 6:** Results for time- and frequency-domain features of EMG data

Parameter	Bipedal stance				Dominant leg stance		Non-dominant leg stance	
	L		R		L	R	L	R
	D	ND	D	ND	D	D	ND	ND
med. gastr.								
AAC (μV)	4.93 (5.30)	3.53 (3.28)	2.60 (1.12)	4.00 (2.85)	15.62 (7.38)	16.32 (6.85)†	13.24 (5.63)	20.40 (8.29)†
DABS_M (μV)	4.93 (5.30)	3.53 (3.28)	2.60 (1.12)	4.00 (2.85)	15.62 (7.38)	16.32 (6.85)†	13.24 (5.63)	20.40 (8.29)†
ENH_WL	2.40 • 10^5^ (1.61 • 10^5^)	1.98 • 10^5^ (1.09 • 10^5^)	1.65 • 10^5^ (0.38 • 10^5^)	2.11 • 10^5^ (0.91 • 10^5^)	5.3 • 10^5^ (1.83 • 10^5^)	5.3 • 10^5^ (1.79 • 10^5^)†	4.8 • 10^5*^ (1.42 • 10^5^)	6.6 • 10^5^ (1.91 • 10^5^)*†
INT_EMG (μV)	1.10 • 10^5^ (1.28 • 10^5^)	7.79 • 10^5^ (8.45 • 10^5^)	5.39 • 10^5^ (3.10 • 10^5^)	9.40 • 10^5^ (8.00 • 10^5^)	4.1 • 10^5^ (2.01 • 10^5^)	4.5 • 10^5^ (2.30 • 10^5^)†	3.5 • 10^5*^ (1.33 • 10^5^)	5.7 • 10^5^ (2.31 • 10^5^)*†
LOG_TK_EO	15.30 (1.81)	14.74 (1.50)	14.57 (1.10)	15.23 (1.52)	18.36 (1.05)	18.34 (1.24)†	18.14 (0.66)	18.84 (1.06)†
MFL (μV)	3.21 (0.38)	3.09 (0.32)	3.06 (0.23)	3.20 (0.32)	3.86 (0.23)	3.86 (0.27)†	3.82 (0.14)	3.97 (0.23)†
WL (μV)	4.93 • 10^5^ (5.30 • 10^5^)	3.53 • 10^5^ (3.28 • 10^5^)	2.60 • 10^5^ (1.12 • 10^5^)	4.00 • 10^5^ (2.85 • 10^5^)	15.6 • 10^5^ (7.38 • 10^5^)	15.7 • 10^5 ^ (6.95 • 10^5^)†	13.2 • 10^5^ (5.63 • 10^5^)	20.4 • 10^5^ (8.29 • 10^5^)†

* between-group differences during dominant/non-dominant leg stance (*p* < 0.05); † within-group differences between dominant and nondominant leg stance (*p* < 0.05)

For further details on the analyzed parameters, please see Table 3.

Values are mean (SD) of each variables. Only those EMG variables are included in which significant difference were found between or within the groups during bi- or unilateral stance. Note that ENH_WL and LOG_TK_EO are unitless.

AAC, Average Amplitude Change; DABS_M, Difference Absolute Mean Value; ENH_WL, Enhanced Wavelength; INT_EMG, Integrated EMG; LOG_TK_EO, Log Teager Kaiser Energy Opertor; MFL, Maximum Fractal Length; WL, Waveform Length.

### Single leg stance

No group and laterality main effects or their interactions was detected by *MANOVA* in any of the kinetic data during single leg stance suggesting that standing stability detected by a variety of *CoP*-related parameters did not differ between *R* and *L* (Table 4).

**FIGURE 2:**
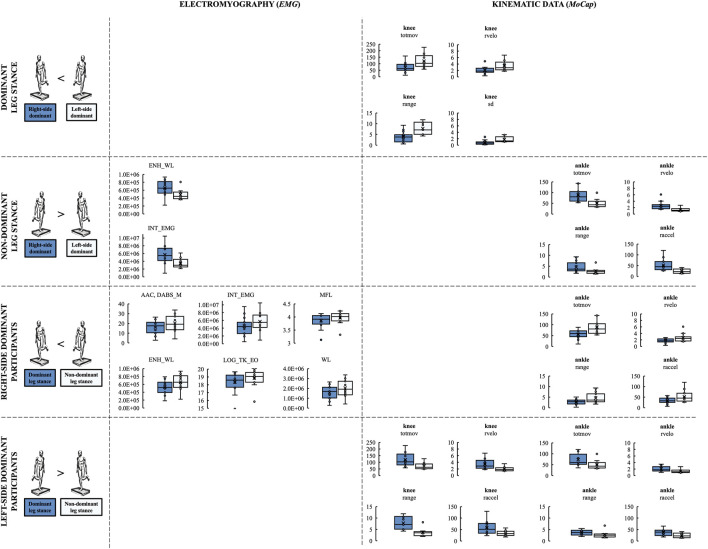
Between-group and within-group differences in CoP, EMG, and MoCap outcomes during unilateral stance. *EMG* outcomes: *AAC*, Average Amplitude Change; *DABS_M*, Difference Absolute Mean Value; *ENH_WL*, Enhanced Wavelength; *INT_EMG*, Integrated EMG; *LOG_TK_EO*, Log Teager Kaiser Energy Opertor; *MFL*, Maximum Fractal Length; *WL*, Waveform Length. Kinematic outcomes: *raccel*, RMS acceleration; *range*, Range; *rvelo*, RMS velocity; *sd*, Standard deviation; *totmov*, Total movement.

There was however a group by laterality interaction in knee and ankle flexion (each Pillai’s trace = 0.286, F_6,39_ = 2.6, *p* = 0.032, η_p_^2^ = 0.29) revealed by *MANOVA*. Additional mixed *ANOVA*s in single kinematic parameters of the knee joint detected group × laterality interaction on *totmov* (F_1,22_ = 6.6, *p* = 0.018, η_p_^2^ = 0.23) (Figure 2) and *range* (F_1,22_ = 8.9, *p* = 0.007, η_p_^2^ = 0.29) of knee flexion with the post-hoc analysis showing larger values in *L* vs *R* (each *p* ≤ 0.022) (Table 5) during dominant leg stance. In addition, this pattern was also found in *rvelo* (*L*: 3.45 ± 1.62°/sec vs *R*: 2.09 ± 1.08°/sec, *p* = 0.027, d = 1.04) and *sd* (*L*: 1.75 ± 0.90° vs *R*: 0.84 ± 0.63°, *p* = 0.005, d = 1.39) of knee flexion during dominant leg stance (Figure 2; Table 5). Moreover, *L* showed larger values in each measured variable of knee flexion when standing on their dominant vs non-dominant leg (each *p* ≤ 0.013). Regarding the kinematic data of ankle flexion, group by laterality interactions were found on *totmov* (F_1,22_ = 13.6, *p* = 0.001, η_p_^2^ = 0.38), *range* (F_1,22_ = 7.0, *p* = 0.015, η_p_^2^ = 0.24), *rvelo* (F_1,22_ = 11.9, *p* = 0.002, η_p_^2^ = 0.35), and *raccel* (F_1,22_ = 17.3, *p* < 0.001, η_p_^2^ = 0.44) of ankle joint angle during non-dominant leg stance with the post-hoc analysis showing larger values in *R* as compared to *L* during non-dominant leg stance (each *p* ≤ 0.05) (Figure 2; Table 5). Moreover, *R* always produced larger ankle joint angle values during non-dominant vs dominant unilateral stance (each *p* ≤ 0.043), however, this pattern was the opposite in case of *L* showing larger values when standing on their dominant vs non-dominant leg (each *p* ≤ 0.033) (Figure 2; Table 5).

There was a group main effect (Pillai’s trace = 0.810, F_1,22_ = 2.4, *p* = 0.038, η_p_^2^ = 0.81) on medial gastrocnemius *EMG* activity revealed by *MANOVA*. Additional statistical analyses based on group × laterality interaction and their post-hoc analyses revealed that *ENH_WL* and *INT_EMG* values of medial gastrocnemius were higher in *R* vs *L* when standing on their non-dominant leg (*p* = 0.045, d = 0.93; *p* = 0.047, d = 0.94; respectively) (Figure 2; Table 6). In addition, *R* had larger values in seven *EMG* features of medial gastrocnemius when standing on the non-dominant vs dominant leg (each *p* ≤ 0.05) (Figure 2; Table 6).

### Association between dependent variables

Strong association was found only among *MoCap* variables that showed between-group differences during non-dominant unilateral standing (each r ≥ 0.778 and *p* < 0.001) but no association was found with medial gastrocnemius *EMG* activity. Similarly, no associations were found between those *MoCap* and *EMG* variables that showed larger values for *R* when they were standing on their non-dominant vs dominant leg (all *p* ≥ 0.05).

## Discussion

We examined the effects of side-dominance on the laterality of bi- and unilateral standing stability in healthy adults by using a multitude of biosignal processing methods. While no differences occurred between left- and right-side dominant participants in kinetic, kinematic, or *EMG* outcomes during bipedal stance, the symmetry index revealed that 87% of right- but only 13% of left-side dominant participants had greater weight-bearing on their non-dominant leg. Regarding single leg stance, larger knee but lower ankle joint kinematic values appeared in left vs right-sided participants during non-dominant stance. Left-vs right-sided participants also had lower medial gastrocnemius *EMG* activation during non-dominant stance. While right-side dominant participants always produced larger values for kinematic data of ankle joint and medial gastrocnemius *EMG* activation during non-dominant vs dominant unilateral stance, this pattern was the opposite for left-sided participants showing larger values when standing on their dominant vs non-dominant leg, i.e., participants had a more stable balance when standing on their right leg.

### Less stable standing balance of right-side dominant participants during non-dominant leg stance

In the present study, participants performed both bi- and unilateral stances, however, data acquired during unilateral stance might be a better marker of postural control because standing on one vs two legs poses a greater challenge for the neural command to organize kinematics and kinetics of stability and could thus be more informative about the role of proprioception in standing stability. Furthermore, unilateral vs bilateral stance challenges the postural control system and is more often performed during daily and sports activities (Vuillerme et al., 2001; Paillard et al., 2006).

Previous studies (Dietz et al., 1989; Goldie et al., 1989; Geurts et al., 1993; Hoffman et al., 1998) have demonstrated no significant differences in postural control measures between healthy participants’ right and left limbs during unilateral stance. However, only one of these studies (Hoffman et al., 1998) determined the functionally dominant and non-dominant lower limb of participants. In the present study, we recruited healthy participants without a history of orthopedic or neuromuscular injuries. Although some previous studies (Freeman et al., 1965; Hoffman et al., 1998) suggested an acute or chronic injury when asymmetry is present in unilateral balance testing, neurodevelopmental studies (Pompeiano, 1985; Previc, 1991) suggest laterality effects on standing posture, i.e., the left leg subserves postural tasks while the right leg performs tasks involving voluntary movement. Differences in the movement characteristics between the right and left limbs are also supported by the association found between lower limb laterality and different activation characteristics in the primary sensorimotor cortex and the basal ganglia (Kapreli et al., 2006). Consequently, it may be not surprising that the left leg is suggested to be the preferred limb for tasks of unipedal stability (Maki, 1991).

Considering that feedback from lower extremity proprioceptors shapes postural strategy while standing (Allum et al., 1998), results from previous studies using *JPS* measurements for the determination of proprioceptive acuity may also serve important information about the laterality effects on standing posture. The existing literature suggests that right-side dominant individuals consistently sense movements more accurately in both upper and lower extremity joints of the non-dominant left vs the right-dominant side (Roy and MacKenzie, 1978; Kurian et al., 1989; Nishizawa, 1991; Goble et al., 2006; Goble and Brown, 2007, 2008; Han et al., 2013; Negyesi et al., 2019). Therefore, we hypothesized that right-side dominant participants would have more stable standing balance when they stand on their non-dominant left vs right-dominant leg. We also expected right vs left-sided participants to have more stable standing balance during non-dominant leg stance.

Numerous parameters can characterize postural performance (Yamamoto et al., 2015). Traditional parameters are based on the motion and velocity of the *CoP*, usually decomposed along the *AP* and *ML* directions. Therefore, we chose to analyze a wide variety of *CoP* data (Table 1). However, interpretations of these parameters differ. Although high postural sway, computed as the total length of the *CoP* path, is often correlated with low postural stability (Paillard and Noé, 2015), reduced sway and tightening of the motion may also constitute a mechanism to handle fear of falling (Adkin et al., 2002). Average *CoP* velocity has been proposed as a better indicator of postural stability than *CoP* displacement since joint velocities are directly used by the body as postural feedback (Masani et al., 2003). Non-linear approaches, such as fractal analyses, can highlight subtle changes in the postural strategy that are not detected by the traditional linear analyses of sway and *CoP* velocity (Doyle et al., 2004; Noda and Demura, 2006). Notably, these non-linear analyses can assess the adaptability of a system, i.e. its capability to efficiently react to external perturbations, however, they have been scarcely applied to quantify the effects of footwear on postural control (Hausselle et al., 2021). Nevertheless, in the present study, none of the *CoP* measures differed significantly between left- and right-side dominant participants’ dominant and non-dominant leg during bipedal or unipedal stance. Overall, the predictive power of *CoP* metrics is questionable considering that perhaps *CoP* path data are not sensitive enough for detecting leg differences in standing stability according to previous studies using unilateral (Matsuda et al., 2008; Lopez-Fernandez et al., 2020) and bipedal (Haddad et al., 2011) stance conditions.

Kinematic data acquired by *MoCap* analysis revealed larger knee but lower ankle joint kinematic values for left vs right-sided participants during non-dominant stance (Table 5). This finding suggests that left and right-side dominant participants use a different balancing strategy while standing on their non-dominant leg. The lower ankle joint angular velocity, acceleration, range, and total movement converged and revealed concordant patterns with the *EMG* activity of their medial gastrocnemius (Table 6). These data are in line with the literature considering that the medial gastrocnemius muscle contributes to maintaining balance during unilateral stance (Tokuno et al., 2007; Hodson-Tole et al., 2013; Lima et al., 2014) and is recruited during low-intensity tasks, i.e., quiet standing (Jacono et al., 2004). Overall, our results suggest that left vs right-sided participants had more stable standing balance while standing on the non-dominant leg.

The larger medial gastrocnemius muscle activation and kinematic values of the ankle joint angle were also present when right-side dominant participants were standing on their non-dominant left vs right-dominant leg. These data suggest worse postural stability of right-as compared to left-side dominant participants during non-dominant leg stance and also compared to their own biosignal data acquired when they stood on their dominant leg. On the other hand, the lack of association between *EMG* data of medial gastrocnemius and the kinematic data of the ankle suggests that the larger activation of medial gastrocnemius muscle was not present due to the larger rotational movements in the ankle joint. In the present study, the activity of triceps surae was measured by the medial gastrocnemius muscle of each leg because this muscle plays a crucial role in controlling posture (Krishnamoorthy et al., 2004). However, because the soleus is also activated to control upright standing, it might have been insightful to determine its activation in left- and right-side dominant participants’ dominant and non-dominant legs. The human soleus and gastrocnemius muscles differ in many respects. The soleus is monoarticular and the gastrocnemius is biarticular. The soleus consists of ∼88% of slow-twitch muscle fibers and the gastrocnemius has ∼52% of fast-twitch muscle fibers (Johnson et al., 1973; Buchthal and Schmalbruch, 1980). Moreover, the control of soleus and gastrocnemius during gait tasks seems to belong to different task groups (Duysens et al., 1991). Thus, it was suggested that despite sharing a common distal tendon, these two muscles may have distinct functional roles (Héroux et al., 2014). Therefore, it is possible that each could affect the knee and/or ankle joint function differently in left-vs right-side dominant participants. Future studies should clarify this idea.

### Less stable standing balance of left-side dominant participants during dominant leg stance

Because neurodevelopmental studies (Pompeiano, 1985; Previc, 1991) indicate left-side dominance for postural control, we expected that right hemisphere specialization may underlie proprioceptive feedback (Naito et al., 2005; Goble and Brown, 2007, 2008) not only in right but also in left-side dominant participants. This idea was also supported by findings of our previous study (Galamb et al., 2018), i.e., left-sided participants performed a target-matching task more accurately with their dominant left vs right knee joint. Therefore, we hypothesized that left-sided participants may have more stable standing balance in their dominant as compared to their non-dominant leg and also as compared to right-side dominant participants during dominant-leg stance. However, our results indicated left vs right-sided participants to have worse markers of balance i.e., larger range and total movement of knee joint (*L*: 7.76 ± 2.92°, *R*: 3.91 ± 2.38°; *L*: 120.64 ± 53.94°, *R*: 71.8 ± 36.98°; respectively) during dominant leg stance. Furthermore, larger values of kinematic data for knee joint angles were present when they stood on their dominant vs non-dominant leg consistently suggesting a more stable standing balance of left-side dominant participants during non-dominant leg stance (Table 5). However, considering that no differences in *CoP* or *EMG* data were found, the practical significance of the kinematic data is questionable especially due to the relatively low sample size. Future studies should recruit more strongly left-side dominant participants to increase the statistical power and to reveal whether between- and within-group differences could also be found in *CoP* and *EMG* data, or in line with the results of previous studies (Tokuno et al., 2007; Hodson-Tole et al., 2013; Lima et al., 2014), no differences can be found between the dominant and non-dominant limb of left-side dominant participants during unilateral stance.

### Laterality effects on *EMG* and kinematic data during bipedal stance

Although unilateral stance is considered to be a better marker of postural control than bipedal upright standing (Vuillerme et al., 2001; Paillard et al., 2006), we also examined left- and right-side dominant participants’ balance stability during bipedal stance. It might be possible that during bipedal stance, the non-dominant leg unknowingly bears a greater portion of body weight hence determines standing stability which in turn would result in a larger *CoP* path, higher muscle activity, or greater range of motion of lower limb joints. However, no differences between left- and right-side dominant participants’ kinetic, kinematic, or *EMG* data was observed during bipedal stance. On the other hand, our results indicate that this hypothesis may apply to right-side dominant participants only considering that 87% of *R* but only 13% of *L* had greater weight-bearing on their non-dominant leg. Nevertheless, we found no differences in any outcomes between participants’ dominant and non-dominant legs during bipedal stance, regardless of group.

### Limitations and future perspectives

One limitation of the present and all other studies considering laterality is the difficulty in the determination of side-dominance due to the contradictory results of functional laterality. For example, we recruited only strongly left- and strongly right-side dominant participants with a laterality index >0.9 according to the results of the Edinburgh Handedness Inventory (Oldfield, 1971) and a questionnaire for determining leg dominance (Spry et al., 1993), still, we expected right-sided participants to have more stable standing balance during non-dominant unilateral stance considering the proposed hemispheric lateralization of proprioception shown in previous neurodevelopmental (Pompeiano, 1985; Previc, 1991), neuroanatomic (Bohannon et al., 1986; Perennou et al., 2008; Duclos et al., 2015) and *JPS* (Roy and MacKenzie, 1978; Kurian et al., 1989; Nishizawa, 1991; Goble et al., 2006; Goble and Brown, 2007, 2008; Han et al., 2013; Negyesi et al., 2019) studies. This leads to the conclusion that hand and/or leg dominance could be defined based on given tasks of questionnaires but we expect this determination not to be universal across tasks. Future studies should consider whether they continue to determine side-dominance based on available questionnaires or rather define the participants’ dominant limb before the main experiment based on their performance in the task of interest. Also, researchers should be careful with the direct interpretation of *JPS* results on proprioception considering that perceptual judgments may not accurately reflect how proprioceptive signals are processed and interpreted (Brown et al., 2003; Filimon et al., 2013) or how they are linked to function (Héroux et al., 2022).

Second, we placed the bipolar EMG electrodes over each muscle belly which might not be the most sufficient method to describe overall muscle activity accurately. Future studies should provide a more comprehensive overview of muscle activity using high-density surface EMG (HD-EMG) (Drost et al., 2006) to reveal regional distribution (e.g., proximal vs distal) of muscle activity during bi- and unilateral stances. Future studies should also consider analyzing biosignal data not only in a conventional way but also using automated, intelligent, and flexible AI-based analytical procedures that solve the difficulty of discovering patterns that do not conform to the expected structure (Chandola et al., 2009). For example, anomaly detection of time series data is widely used in biomedical analyses to detect abnormal ECG (Soga et al., 2021) or EEG (Takahashi et al., 2020). We could not perform such an analysis due to our experimental setup considering that healthy participants were instructed to stand quietly and only successful trials were taken into the analyses. Future studies recruiting both healthy participants and patients with orthopedic or neuromuscular disorders should gather long-lasting time-series data with unexpected events, i.e., falls, balance problems when standing up, sitting down, or even losses of balance during walking to label the data for efficient anomaly detection. Detecting the underlying mechanism of the interaction between side-dominance and posture could serve as the basis for developing more efficient rehabilitation strategies after an injury or even after left- or right-hemisphere damage.

Overall, our results suggest that side-dominance affects biomechanical and neuromuscular control strategies during unilateral standing, which may have implications for the understanding of mechanisms for rehabilitation.

## Data Availability

The datasets generated during and analyzed during the current study are not publicly available due to the large amount of ground reaction force, EMG, and motion capture data with associated excel files but are available from the corresponding author on reasonable request.

## Abbreviations

ANOVA, analysis of variance; AP, anterior-posterior; area_ce, 95% confidence ellipse area; area_sw, sway area; CoP, center of pressure; CNS, central nervous system; EMG, electromyography; FD, fractal dimension; GRF, ground reaction force; JPS, joint position sense; mdist, mean distance; mfreq, mean frequency; mvelo, mean velocity; ML, mediolateral; MoCap, motion capture; mpi, mean percentage index; rms, root mean square; raccel, root mean square acceleration; rvelo, root mean square velocity; SampEn, sample entropy; sd, standard deviation of MoCap data; SD, standard deviation of CoP; totmov, total movement.
